# Integrated engineering of enzymes and microorganisms for improving the efficiency of industrial lignocellulose deconstruction

**DOI:** 10.1016/j.engmic.2021.100005

**Published:** 2021-10-29

**Authors:** Guodong Liu, Yinbo Qu

**Affiliations:** a State Key Laboratory of Microbial Technology, Shandong University, Qingdao 266237, China; b National Glycoengineering Research Center, Shandong University, Qingdao 266237, China

**Keywords:** Lignocellulose, Cellulase, Hemicellulase, Fungi, Genetic engineering, Biorefinery

## Abstract

Bioconversion of lignocellulosic biomass to fuels and chemicals represents a new manufacturing paradigm that can help address society's energy, resource, and environmental problems. However, the low efficiency and high cost of lignocellulolytic enzymes currently used hinder their use in the industrial deconstruction of lignocellulose. To overcome these challenges, research efforts have focused on engineering the properties, synergy, and production of lignocellulolytic enzymes. First, lignocellulolytic enzymes’ catalytic efficiency, stability, and tolerance to inhibitory compounds have been improved through enzyme mining and engineering. Second, synergistic actions between different enzyme components have been strengthened to construct customized enzyme cocktails for the degradation of specific lignocellulosic substrates. Third, biological processes for protein synthesis and cell morphogenesis in microorganisms have been engineered to achieve a high level and low-cost production of lignocellulolytic enzymes. In this review, the relevant progresses and challenges in these fields are summarized. Integrated engineering is proposed to be essential to achieve cost-effective enzymatic deconstruction of lignocellulose in the future.

## 1 Introduction

Lignocellulosic biomass is the most abundant organic resource on Earth. The major components of lignocellulose include cellulose, hemicellulose, and lignin, which can be enzymatically or chemically degraded into simple sugars and aromatic compounds (Wei et al., 2009). The latter compounds can be further converted into various fuels (e.g., ethanol) and chemicals, which are expected to replace those produced from petroleum (Somerville et al., 2010, Werpy and Petersen, 2004). The plentiful supply and sustainable characteristics of lignocellulose make it the most attractive feedstock in biomass-based refinery (Ragauskas et al., 2006). In addition to helping to alleviate the current resource crisis, the production of lignocellulose-derived fuels and chemicals is also beneficial in the mitigation of greenhouse gases (Field et al., 2020).

The commercialization of lignocellulose biorefinery is still in its infancy. One of the bottlenecks of industrial lignocellulose conversion is the lack of ideal technologies for substrate deconstruction (Chandel et al., 2018). While enzymatic deconstruction has the advantages of being specific, easy to operate, and eco-friendly, the required dosage of lignocellulolytic enzymes is high (∼10–15 g/kg substrate) due to their low efficiencies (Klein-Marcuschamer et al., 2012). In the past 20 years, the performance of enzymes in lignocellulosic biomass saccharification has been significantly improved with the development of new-generation cellulase preparations by leading enzyme companies (McMillan et al., 2011). Using the optimized enzymes, several commercial and precommercial plants have been built for cellulosic ethanol production (Hortsch and Corvo, 2020). However, the cost of enzymes is still high compared with the well-developed starch saccharification process (Ellilä et al., 2017, Liu et al., 2016). Therefore, the production of low-cost and effective lignocellulolytic enzymes remains a major focus of research to achieve a more economic conversion of lignocellulosic biomass to fuels and chemicals.

**Fig. 1:**
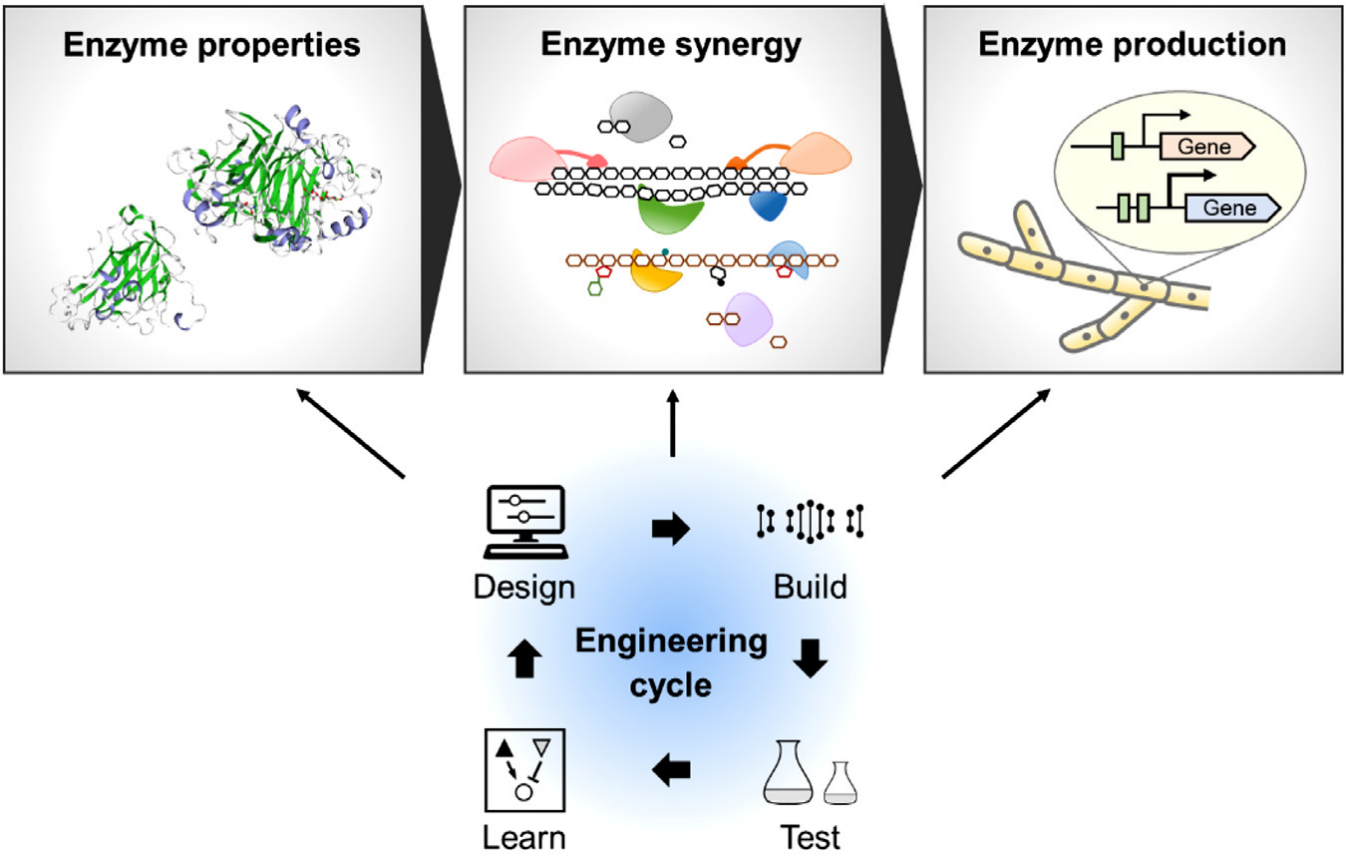
Bioengineering approaches for the development of cost-effective lignocellulolytic enzymes. The Design-Build-Test-Learn cycle (Nielsen and Keasling, 2016) can be used in multiple levels of engineering in this respect.

Several strategies can be used to lower the cost of enzymes for lignocellulose deconstruction (Liu et al., 2013a). First, improvement in enzymes’ efficiency could reduce their dosage. Second, strain engineering and fermentation optimization could reduce the cost of enzyme production. Third, on-site enzyme production in biorefinery plants could minimize the cost of enzyme transport and storage (Johnson, 2016). To achieve these goals, lignocellulolytic enzyme molecules, enzyme systems, and their production hosts need to be effectively engineered and integrated (Fig. 1) (Contreras et al., 2020). This review summarizes the progress witnessed in engineering lignocellulolytic enzymes and enzyme-producing microorganisms and presents future perspectives in the context of developing cost-effective lignocellulose conversion processes.

## 2 Lignocellulolytic enzymes

All the major components in lignocellulose have specific compositional and structural characteristics, which ensure biological recalcitrance. Therefore, it is not surprising that complex enzyme systems are needed for the degradation of lignocellulose in natural living systems. So far, more than 30 classes of enzymes produced by microorganisms have been found to be involved in the deconstruction of lignocellulose (Liu et al., 2013a, van den Brink and de Vries, 2011). Of note, in addition to canonical hydrolytic enzymes, lytic polysaccharide monooxygenases (LPMOs) and several other oxidoreductases play important roles in lignocellulosic degradation (Bissaro et al., 2018).

### 2.1 Cellulolytic enzymes

Cellulose is a polysaccharide composed of linear β-1,4-glucan chains, which form crystalline and amorphous structures. In the crystalline region, glucan chains are held together via hydrogen bonds and hydrophobic interactions (Beckham et al., 2011), which are hard to access and break by enzymes. Typically, endo-β-1,4-glucanases (EGs, EC number 3.2.1.4) and cellobiohydrolases (CBHs, 3.2.1.176 and 3.2.1.91, acting on reducing or non-reducing ends, respectively) synergistically hydrolyze cellulose chains to cello-oligosaccharides, which are further hydrolyzed to glucose by β-glucosidases (3.2.1.21). Cellulose-active LPMOs (1.14.99.54 and 1.14.99.56) break the β-1,4-glycosidic bonds in cellulose and release cello-oligosaccharides containing d-glucono-1,5-lactone or 4-dehydro-d-glucosyl residues (Hemsworth et al., 2015, Frandsen et al., 2016). CBHs, LPMOs, plant expansin-like proteins (e.g., swollenin in fungi), and some EGs are able to attack the crystalline structure of cellulose (Teeri et al., 1998, Eibinger et al., 2017, Zhang et al., 2021, Sakon et al., 1997) and thus have become engineering targets for the degradation of natural cellulose.

### 2.2 Hemicellulolytic enzymes

Hemicelluloses are complex and heterogeneous polysaccharides formed by a set of carbohydrate and non-carbohydrate residues (Scheller and Ulvskov, 2010, Willför et al., 2003). The content, composition, and structure of hemicellulose in plant biomass are dependent on the species, tissue type, and developmental stage. The recalcitrance of hemicellulosic biomass is largely attributable to the need for a complete enzyme system to break various chemical bonds necessary for complete deconstruction. Similar to what is observed in cellulases, synergistic effects can be observed between different hemicellulolytic enzymes. Particularly, the actions of some backbone-degrading enzymes (e.g., endo-β-1,4-xylanase and endo-β-1,4-mannanase) are sensitive to side-chain substitutions (Pollet et al., 2010, McCleary et al., 1983). Therefore, debranching enzymes such as α-l-arabinofuranosidase, α-glucuronidase, acetyl xylan esterase, and feruloyl esterase are important for the efficient degradation of xylans containing side groups, which are the main hemicelluloses in grasses and hardwoods (de Vries et al., 2000, Wang et al., 2015). Similarly, α-galactosidases that remove galactose side chains can improve the degradation of galactoglucomannan, the dominant hemicellulose in softwoods (de Vries et al., 2000, Wang et al., 2015). In addition, hemicellulose-active LPMOs have been reported to act synergistically with hydrolytic enzymes to efficiently degrade substrates (Couturier et al., 2018).

### 2.3 Pectinolytic enzymes

Pectin, another member of the polysaccharide family of plant cell walls, is structurally more complex than hemicelluloses. Despite its low content in graminaceous monocots, pectin is abundant in dicots and woody biomass (Biswal et al., 2017). Homogalacturonan, a major form of pectin, is a polymer of α-1,4-linked d-galacturonic acid residues, whose side groups can be esterified (Muller-Maatsch et al., 2014). Accordingly, pectin methyl esterase and pectin acetyl esterase hydrolyze the methyl- and acetyl-ester groups, respectively, and polygalacturonase cleaves the de-esterified backbone. In addition, pectin lyases and pectate lyases break down esterified and unmodified pectin, respectively, through a β-elimination mechanism. In contrast, rhamnogalacturonans have rhamnose residues on their backbones or side chains and contain additional kinds of glycosyl residues, whose degradation requires other types of enzymes (Duran Garzon et al., 2021).

### 2.4 Ligninolytic enzymes

Lignins are phenylpropanoid polymers derived from hydroxycinnamyl alcohols and other aromatic monomers (Ralph et al., 2019). The enzymatic deconstruction of major chemical bonds (e.g., β-aryl ether bonds, resinol β-β bonds, and biphenyl bonds) are more difficult compared with the bonds found in cellulose and hemicellulose. Some fungal (e.g., white-rot fungi) and bacterial species can degrade lignin using a set of secreted ligninolytic enzymes, including lignin peroxidases, manganese peroxidases, versatile peroxidases, laccases, and other enzymes (Pollegioni et al., 2015). Nevertheless, these enzymes have not been used for the industrial degradation of lignin to date. One reason for this is that these peroxidases could catalyze both depolymerization and polymerization reactions in lignin (Zeng et al., 2017). Catabolism of lignin's degradation products by bacteria was shown to promote the enzymatic depolymerization process, but the conversion efficiency is still much lower than those of cellulose and hemicellulose (Salvachúa et al., 2016). Chemical or chemo-biological degradation or even the conversion to materials instead of degradation may be more realistic processes for the utilization of industrial lignin in the future. The rest of this article will mainly focus on the enzymes that are capable of degrading cellulose and hemicellulose.

### 2.5 The organization of lignocellulolytic enzymes

**Fig. 2:**
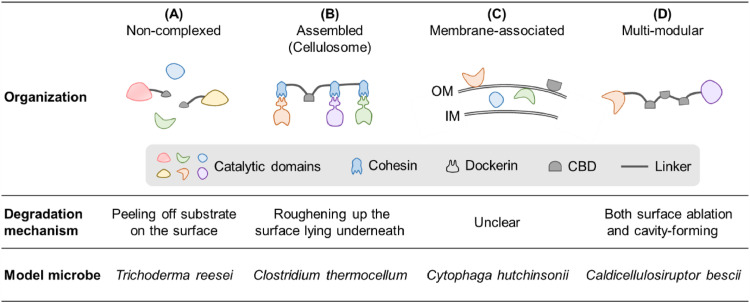
Different forms of cellulolytic enzyme systems in microorganisms. OM, outer membrane; IM, inner cytoplasmic membrane; CBD, cellulose-binding domain. The mechanisms of cellulose degradation were reported in references (Brunecky et al., 2013, Eibinger et al., 2020, Liu et al., 2011, Resch et al., 2013).

Lignocellulolytic enzymes usually act synergistically during degradation, although their organization systems vary among different microorganisms (Cragg et al., 2015). Aerobic fungi and many aerobic bacteria secrete noncomplexed enzymes, with these enzymes containing one single catalytic domain in most cases (Zhang and Lynd, 2004). Frequently, a cellulose-binding domain is connected to the catalytic domain by a flexible linker peptide (Fig. 2**A**). Filamentous fungi (e.g., *Trichoderma reesei* and *Aspergillus niger*) that secrete such “free” enzyme systems are the main producers of industrial lignocellulolytic enzymes (Bischof et al., 2016, Pel et al., 2007). Anaerobic bacteria and fungi can form cellulase complexes (referred to as “cellulosomes”) attached to the cell, in which multiple cellulases are anchored to scaffolding proteins through cohesin–dockerin interactions (Fig. 2**B**) (Bayer et al., 2004, Lillington et al., 2021). The cellulosome-producing *Clostridium thermocellum* has been used as a whole-cell biocatalyst for lignocellulose saccharification (Liu et al., 2019). Aside from these two extensively studied cellulase systems, some bacteria (e.g., *Cytophaga hutchinsonii*) degrade lignocellulose through the combined action of extracellular, outer membrane (and outer membrane vesicle)-associated, and periplasmic enzymes (Fig. 2**C**) (Taillefer et al., 2018, Zhu and McBride, 2017, Arntzen et al., 2017), while some others (e.g., *Caldicellulosiruptor bescii*) secrete noncomplexed cellulases/hemicellulases containing multiple catalytic and cellulose-binding domains (Fig. 2**D**) (Brunecky et al., 2013, Su et al., 2012). The latter two forms of cellulolytic systems are highly active on crystalline cellulose, thus providing alternative strategies for industrial cellulose deconstruction.

## 3 Mining and engineering for better enzymes

**Fig. 3:**
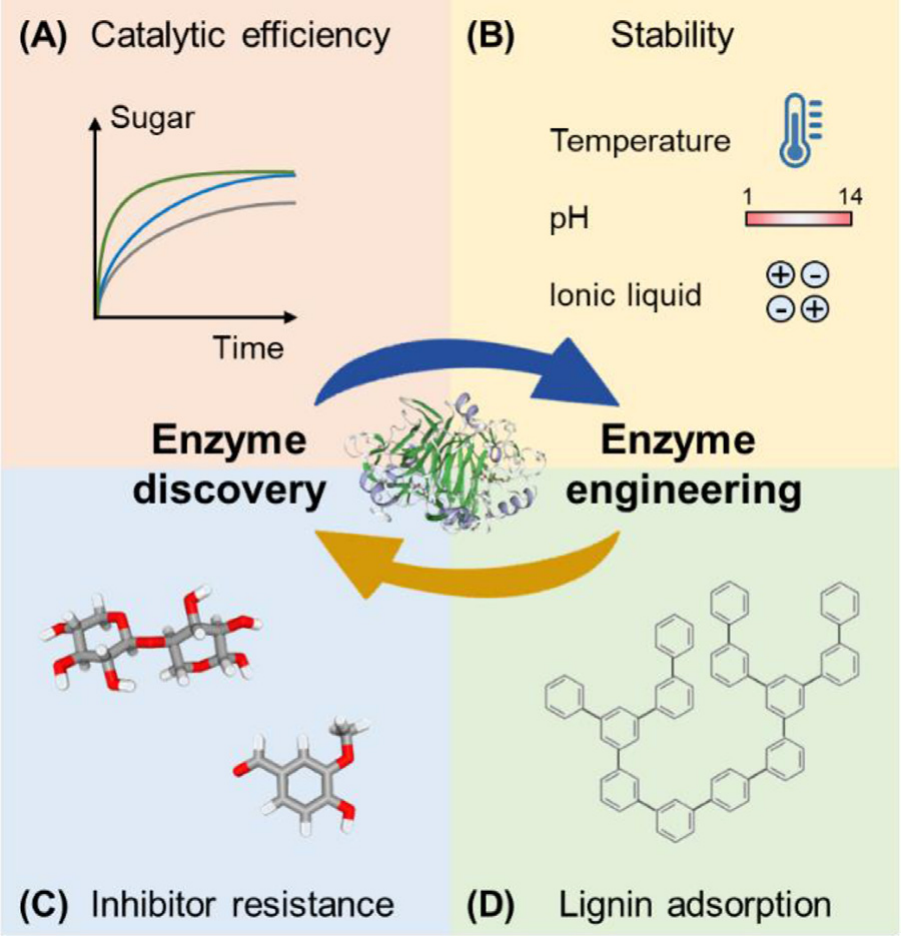
Combination of discovery and engineering approaches to improve the performance of lignocellulolytic enzymes.

The discovery of new enzymes and the engineering of currently used enzymes have been successfully used to improve the performances of lignocellulolytic enzymes. For the discovery approach, sequence-based genome mining and function-based protein separation both have provided enzymes with unique properties (see the following subsections.). For the engineering approach, directed evolution and rational design have been used to improve the properties of many enzymes. In some cases, the two approaches have been combined to effectively generate “superior” enzyme mutants (Fig. 3).

### 3.1 Catalytic efficiency

The major problem of cellulases is their low efficiency in acting on natural crystalline cellulose (Gao et al., 2013). Despite many studies on the molecular mechanisms of substrate binding and catalysis, little information has been used to guide the engineering of cellulolytic enzymes. In fungi, CBH I is the key enzyme for crystalline cellulose hydrolysis. The most effective strategy for engineering this enzyme to date has come from studying the enzyme's diversity in nature. For example, CBH I and several other cellulases from *Penicillium verruculosum* have shown higher hydrolytic efficiencies than those from *T. reesei* (Morozova et al., 2010). Based on the comparison with CBH I from *Penicillium funiculosum*, which revealed a 60% higher activity than CBH I from *T. reesei*, the performance of the latter enzyme was enhanced via domain replacements or the precise mutation of two sites in its catalytic domain (Taylor et al., 2018). For *Penicillium oxalicum*’s CBH I, replacement of the non-catalytic region with the corresponding sequence in *T. reesei* also improved its activity on crystalline cellulose (Du et al., 2018). Inspired by the multimodular architecture of the cellulase CelA from *C. bescii* (Fig. 2**D**), a chimeric cellulase containing EG and cellulose-binding domains from bacteria and a CBH domain from fungi was constructed. The chimeric enzyme showed enhanced activity compared with noncomplexed cellulases (Brunecky et al., 2020). In addition, rational design was used to improve the activity of an EG by reducing product inhibition (Baker et al., 2005). Using these engineered lignocellulolytic enzymes to replace native enzymes in industrial host strains is expected to produce new cellulolytic enzyme systems with higher efficiencies. Indeed, the replacement of the native *cbh1* gene in *T. reesei* with its homolog from *Chaetomium thermophilum* led to a 2.2-fold increase in overall cellulase activity (Jiang et al., 2020).

### 3.2 Stability

The degradation of lignocellulose by current industrial cellulolytic cocktails is usually operated at 45°C–50°C. Improving the thermostability of lignocellulolytic enzymes allows for hydrolysis at elevated temperatures, which is expected to have the advantages of high hydrolysis efficiency, low viscosity and low risk of microbial contamination. Many thermostable lignocellulolytic enzymes have been isolated from thermophilic microorganisms (Berka et al., 2011, Voutilainen et al., 2008). A reconstituted enzyme system using three thermostable cellulases and a thermostable xylanase has shown comparable or even better performances in the hydrolysis of pretreated raw materials at 60°C compared with those of *T. reesei*’s enzymes at 45°C (Viikari et al., 2007). In addition, protein engineering (e.g., structure-guided recombination) was able to produce artificial cellulases of higher thermostabilities (Heinzelman et al., 2009, Heinzelman et al., 2010). The stabilities of enzymes under specific environments are also an engineering focus considering the integration of biomass saccharification with other processes. For example, the tolerance of lignocellulolytic enzymes to ionic liquids needs to be improved when ionic liquids are used for biomass pretreatment (Wolski et al., 2016).

### 3.3 Inhibitor resistance

Lignocellulolytic enzymes can be inhibited or inactivated by many compounds produced during the pretreatment and saccharification of biomass. The inhibitors include sugars, sugar derivatives, and phenolic compounds (Jing et al., 2009, Kim et al., 2011). For example, 2 mg/ml of tannic acid inhibited up to 60% of the cellulase activity of a commercial cellulase preparation from *T. reesei* (Ximenes et al., 2011). Inhibition by some products can be alleviated by supplementing with corresponding hydrolases (see Section 4) or by the simultaneous fermentation of sugars to downstream products. However, some inhibitors (e.g., monomeric phenolics) are difficult to remove, and therefore, the improvement of enzymes’ resistance is essential to achieve efficient degradation. The β-glucosidase from *Aspergillus niger* was shown to be more resistant to phenolics than that from *T. reesei*, suggesting the possibility of improving phenolic resistance by enzyme discovery and engineering (Ximenes et al., 2011). To our knowledge, such engineering work has not been reported for lignocellulolytic enzymes.

### 3.4 Lignin adsorption

Cellulases and hemicellulases can be adsorbed to lignin through hydrophobic and electrostatic interactions, which inhibit the hydrolysis performed by these enzymes. Cellulases produced by some fungi were found to be less inhibited by lignin than cellulases from *T. reesei*, whose mechanisms remain unclear (Berlin et al., 2006). Fungal cellulases’ non-productive binding to lignin was mainly attributed to their cellulose-binding domains (Palonen et al., 2004). Nevertheless, engineering cellulose-binding domains to inhibit lignin binding is challenging, since the amino acid residues involved in lignin binding also play roles in cellulose-binding (Strobel et al., 2015). The combined engineering of cellulose-binding domain (adding negative charges) and linker peptide (adding *O*-glycosylation sites) generated a mutant with a 2.5-fold reduction in lignin affinity but a less change in cellulose affinity, which produced 40% more glucose from raw lignocellulosic biomass than the parent enzyme (Strobel et al., 2016, Lu et al., 2017). In addition, the catalytic domain of some lignocellulolytic enzymes may also be involved in lignin adsorption (Rahikainen et al., 2013), which should be evaluated on a case-by-case basis.

### 3.5 Challenges in the engineering of lignocellulolytic enzymes

Engineering lignocellulolytic enzymes faces a number of challenges. First, many enzymes are expressed in commonly used protein production hosts (e.g., *Escherichia coli* and *Saccharomyces cerevisiae*) for characterizations. However, these hosts have different glycosylations and N-terminal modifications from industrial hosts (e.g., *T. reesei*), which could significantly affect enzymes’ properties (Chokhawala et al., 2015, Jeoh et al., 2008). Second, substrate derivatives and analogs (e.g., nitrophenyl glycosides) are convenient for the high-throughput screening of lignocellulolytic enzymes, but the measured activities may not represent those on real lignocellulosic biomass. As an example, two α-l-arabinofuranosidases of similar activity on *p*-nitrophenyl-l-arabinofuranoside show up to a seven times difference in their hydrolysis efficiency of arabinoxylan (Shinozaki et al., 2014). Third, studies have reported trade-offs between the enzymes’ properties (e.g., stability and activity) in the process of engineering lignocellulolytic enzymes (Heinzelman et al., 2010, Borisova et al., 2018). Finally, different biorefinery processes have different and specific requirements to engineer enzymes. Different from separated hydrolysis and fermentation, the simultaneous saccharification and fermentation process is able to overcome the challenge of product inhibition by consuming saccharification products but requires the enzymes to act efficiently under specific fermentation conditions (e.g., temperature and pH) (Öhgren et al., 2007). Taken together, the performance of “better” lignocellulolytic enzymes should be evaluated or confirmed in the context of industrial hosts, natural substrates and realistic application conditions.

## 4 Construction of high-performance enzyme cocktails

The synergistic cooperation of cellulases and hemicellulases of different substrate specificities is a key element to be considered in the engineering steps beyond the engineering of individual enzymes (Fig. 1) (Van Dyk and Pletschke, 2012). Based on the understanding of natural lignocellulolytic enzyme mixtures, achievements have been made in the design of artificial enzyme cocktails for the more efficient degradation of specific substrates.

### 4.1 Types of enzyme synergy

Generally, the synergy between lignocellulolytic enzymes can be linked by two mechanisms: an increase in the accessibility of a substrate and the prevention of enzymes’ inhibition. For example, LPMOs enhance the action of hydrolytic enzymes by disrupting the tight structure of crystalline cellulose (Song et al., 2018), and debranching enzymes promote the degradation of hemicelluloses by increasing their accessibility to backbone-degrading enzymes (see Section 2.2.). In addition, the synergy of some cellulases is believed to come from the liberation of stalled enzymes through the action of other cellulases (Igarashi et al., 2011). For the second mechanism, the supplementation of enzymes acting on oligosaccharides, sugar lactones, and oligomeric phenolics can mitigate their inhibition of lignocellulolytic enzymes and therefore improve their degrading efficiency (Qing and Wyman, 2011, Peng et al., 2017, Tejirian and Xu, 2011, Igarashi et al., 1998). It should be emphasized that synergistic action can occur between enzymes acting on different substrates. For example, supplementation of hemicellulases or pectinases is beneficial for the degradation of cellulose through the removal of physical blocks and/or the elimination of oligosaccharides that inhibit cellulases’ activities (Hu et al., 2011, Yang et al., 2017, Beri et al., 2021, Kumar and Wyman, 2014, Spagnuolo et al., 1997).

Also notable is the synergy between cellulase systems of different organizations. A comparative study revealed that *C. thermocellum*’s cellulosomal system was more efficient in the degradation of crystalline cellulose than the fungal free enzyme system. Strikingly, the two systems, utilizing different mechanisms of degradation, showed significant synergy in the hydrolysis of a pure cellulose substrate (Resch et al., 2013). Whether such synergistic effects also occur on complex biomass and the possible cooperation between other types of cellulase systems (Fig. 2) are both areas worth exploring in the future.

### 4.2 Tailoring enzyme mixtures by improving enzyme synergies

The lignocellulolytic enzyme mixtures produced by naturally isolated microorganisms are adapted to their habitats and nutrition strategies. The compositions of natural enzyme mixtures are usually unbalanced and need modifications for industrial applications (Gusakov et al., 2007, Waghmare et al., 2021). The genome sequencing of *T. reesei* suggested that it lacks genes encoding feruloyl esterase and most kinds of pectinases (Martinez et al., 2008). In contrast, many *Aspergillus* and *Penicillium* species have a comprehensive range of hemicellulolytic and pectinolytic enzymes, which are complementary to *T. reesei*’s enzymes (Coutinho et al., 2009, Liu et al., 2013b). Therefore, strategies to overcome these limitations could focus on combining enzymes from multiple species with complementary functional profiles or integrating heterologous enzymes into the genomes of fungal cell factories to complement missing enzymatic activities. So far, major advancements in the engineering of enzyme mixtures from *T. reesei* include enhancements of β-glucosidase, LPMO, and xylanase (Sun et al., 2015, Chylenski et al., 2017). With these optimizations, the dosage of proteins able to achieve a favorable degrading efficiency can be significantly reduced. For cellulosome produced by *C. thermocellum*, the supplementation of β-glucosidase and hemicellulase also improved their degrading efficiency (Liu et al., 2019, Qi et al., 2021). Furthermore, artificial cellulosomes assembled from lignocellulolytic enzymes of different origins (i.e., “designer cellulosomes”) were shown to perform better than free enzymes (Artzi et al., 2017, Davidi et al., 2016).

The “optimal” composition of a lignocellulolytic enzyme mixture is highly dependent on the characteristics of the substrates involved (Du et al., 2020). Due to our limited knowledge of the structure of complex lignocellulosic substrates, the optimization of an enzyme mixture is often performed in a non-rational manner (Jabbour et al., 2013). Recently, determination of substrates’ structures, particularly that of the recalcitrant components, was used to guide the rational optimization of enzyme mixtures. For example, elucidation of the special structures of glucuronoarabinoxylan's side chains in corn kernel fiber indicated the requirement of a mixture of α-d-xylosidase, β-d-xylosidase, α-l-arabinofuranosidase, and α-l-galactosidase for its complete degradation (Beri et al., 2020).

## 5 Development of efficient enzyme-producing microorganisms

**Fig. 4:**
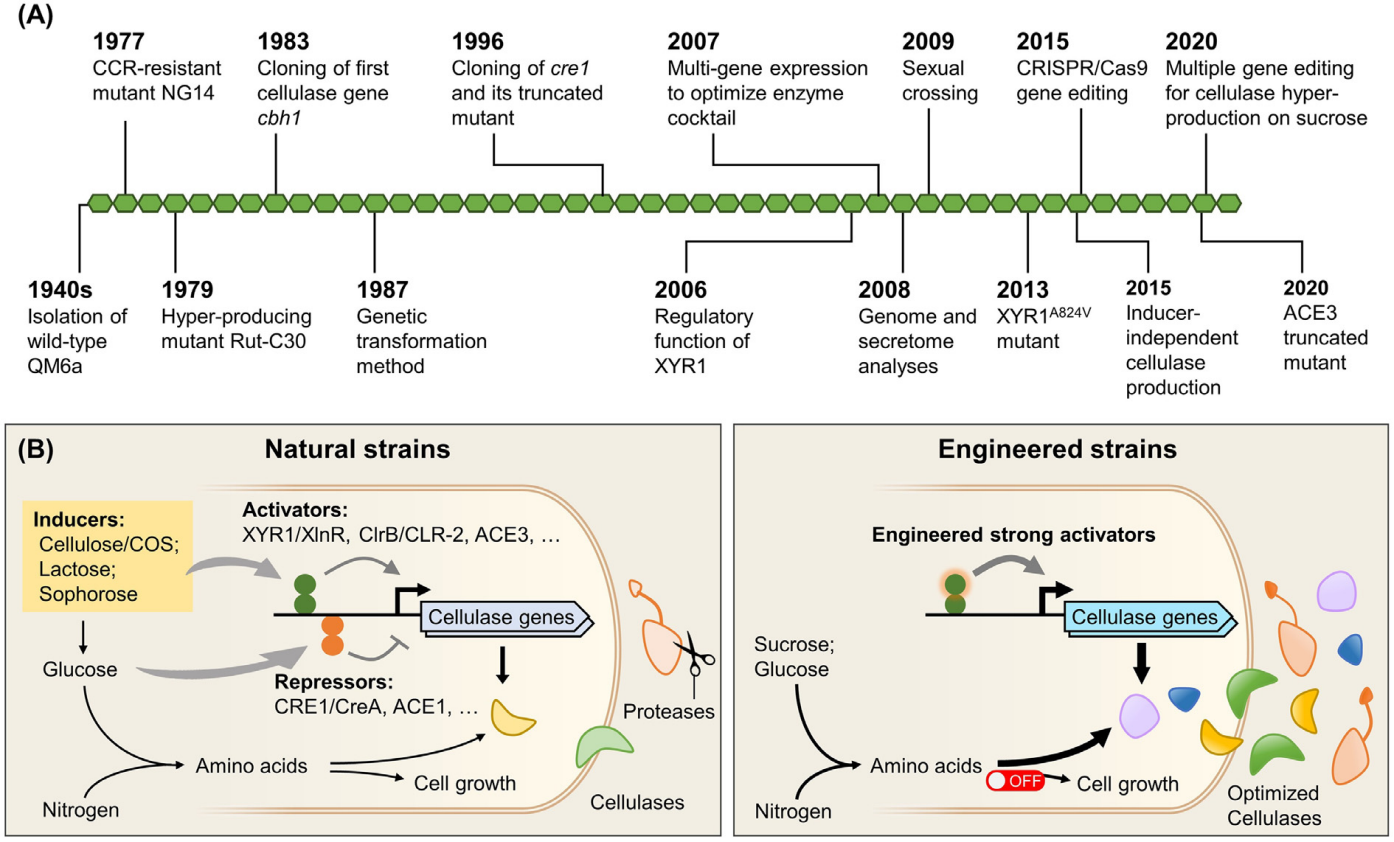
Engineering of filamentous fungi for the production of lignocellulolytic enzymes. (A) Selected milestones in strain improvement of *T. reesei* (Bischof et al., 2016, Allen et al., 2009, Montenecourt and Eveleigh, 1977, Montenecourt and Eveleigh, 1979, Shoemaker et al., 1983, Penttila et al., 1987, Ilmen et al., 1996, Stricker et al., 2006, Merino and Cherry, 2007, Martinez et al., 2008, Herpoel-Gimbert et al., 2008, Seidl et al., 2009, Derntl et al., 2013, Lv et al., 2015, Liu et al., 2015, Chen et al., 2020, Fonseca et al., 2020). For most achievements, the years of corresponding publications are shown. CCR, carbon catabolite repression. (B) Rewiring the cellular networks for cost-effective production of optimized lignocellulolytic enzymes. COS, cello-oligosaccharides.

The lignocellulolytic enzyme mixtures can be applied for industrial applications only when they can be produced with high productivities and at low-cost. As the main producer of cellulase in industry, *T. reesei* has been significantly improved through classical mutagenesis and genetic engineering (Fig. 4**A**). Particularly, the characterization of high-producing mutant strains identified several sequence mutations in transcription factors, and the development of CRISPR/Cas9-based genome editing methods has brought a strong impetus to rational strain engineering. Similar studies have been performed in other lignocellulolytic enzyme-producing fungi, such as *Myceliophthora thermophila* (Visser et al., 2011), *P. oxalicum* (Liu et al., 2013c), and *P. funiculosum* (Randhawa et al., 2021). Nevertheless, the cellular processes for protein synthesis and secretion, as well as the physiology and morphology of cells, require further elucidation in these fungi to further decrease the cost of production. Compared with fungi, genetic engineering of bacteria (e.g., *C. thermocellum*) for higher lignocellulolytic enzyme production is less often reported.

### 5.1 Rewiring the regulatory networks for high level enzyme production

A high level of transcription of the genes encoding lignocellulolytic enzymes is a prerequisite for a high level of production of these enzymes. In wild-type fungal strains, the transcription of lignocellulolytic enzymes is induced by specific inducers (e.g., cellulose, cello-oligosaccharides, lactose, and sophorose for the induction of *T. reesei*’s cellulases) and repressed by readily accessible carbon sources (e.g., glucose) (Foreman et al., 2003). The regulation is mediated by multiple transcription factors, which constitute regulatory networks. Generally, some pathway-specific transcriptional activators (mostly belonging to the Zn_2_Cys_6_ family) are required for the expression of a group of functionally related enzymes in response to environmental lignocellulosic biomass. In addition, other “accessory transcription factors” contribute to but are not essential for the expression of lignocellulolytic enzymes (Huberman et al., 2016). On the other hand, the expression of a variety of lignocellulolytic enzymes and their transcriptional activators are negatively controlled by the carbon catabolite repression (CCR) system.

Studies in different fungal species have revealed both conserved and species-specific regulatory mechanisms that control the expression of lignocellulolytic enzymes. XYR1/XlnR is the major transcriptional activator of cellulase and xylanolytic enzyme expression in *T. reesei* and *A. niger* (Stricker et al., 2006, van Peij et al., 1998), while its functions in *Neurospora crassa* and *P. oxalicum* are mainly involved in the expression of xylanolytic enzymes (Li et al., 2015, Sun et al., 2012, Gao et al., 2017). In the latter two species, another activator CLR-2/ClrB is essential for the expression of cellulolytic enzymes (Li et al., 2015, Coradetti et al., 2012). Also, AraR and GaaR are required for the expression of arabinose- and galacturonic acid-releasing enzymes, respectively, in some *Aspergillus* and *Penicillium* species (Battaglia et al., 2011, Alazi et al., 2016, Gao et al., 2019a). For CCR, CRE1/CreA has been identified as a major regulator that targets a wide range of genes encoding plant biomass-degrading enzymes (Antonieto et al., 2014, Peng et al., 2021).

Lignocellulolytic enzyme-inducing sugars have been widely used as carbon sources for enzyme production; however, insoluble lignocellulosic biomass is not favored by submerged fermentation, while soluble inducers are expensive and/or have limited accessibility (Li et al., 2016). Therefore, rewiring the regulatory network for the expression of lignocellulolytic enzymes is essential for their production on conventional carbon sources like glucose and sucrose (Fig. 4**B**) (Huberman et al., 2016). In *T. reesei*, inactivation of the transcription factor CRE1 alleviated CCR (Ilmen et al., 1996), and overexpression or constitutive activation of activators XYR1 or ACE3 enabled cellulase production with glucose as the sole carbon source (Ellilä et al., 2017, Lv et al., 2015, Luo et al., 2020). The Rut-C30 strain, which already contains beneficial mutations for higher extracellular enzyme production in the genes *cre1* and *ace3*, was further optimized by engineering the transcription factors XYR1 and ACE1. A hypersecreting strain was constructed, producing 80.6 g L^−1^ extracellular protein on sucrose (Fonseca et al., 2020). In *P. oxalicum*, the cellulase gene activator ClrB was constitutively activated through the deletion of a potential regulatory region, which led to cellulase production on glucose (Gao et al., 2019b). In *A. niger*, overexpression of GaaR is enough to trigger an inducer-independent production of pectinases (Alazi et al., 2018). It should be noted that although many transcription factors were found to be involved in lignocellulolytic enzyme expression, their functions may be dispensable when the genetic background is changed (Cao et al., 2017, Yan et al., 2021). Thus, the genetic interactions between regulator genes should be carefully considered in the construction of high-producing enzyme strains.

### 5.2 Metabolic and morphological engineering

The metabolic network is another focus of engineering for the industrial production of lignocellulolytic enzymes (Fig. 4**B**). First, elimination of specific proteases is helpful to diminish the degradation of lignocellulolytic enzymes (Qian et al., 2019). Second, the fermentation time of currently used fungal strains is usually longer than 5 days, which consumes more energy and power than many bacterial and yeast protein hosts. Therefore, improving strains’ growth or protein production rate is expected to reduce production cost. In *T. reesei*, the disruption of genes for sorbicillinoids synthesis was found to improve the yield of cell biomass by around 30% (Derntl et al., 2017). Third, the synthesis of extracellular enzymes and cell biomass compete for precursors (e.g., amino acids) and energy, which may be balanced by dynamic metabolic control (Liu et al., 2018, Lemmerer et al., 2019).

On the other hand, the high viscosity of cultivation broth caused by fungal hyphae remarkably limits mass transfers and oxygen supplies, making the fermentation energy intensive. Indeed, a hyperproducing mutant of *M. thermophila* forms fragmental mycelium and spore-like cells in a liquid medium, resulting in a 50-fold lower viscosity (Verdoes et al., 2007). Recently, the disruption of several genes in *T. reesei* was found to reduce the viscosity during fermentation from 23% to 89% via altering the mycelial morphology (Bodie et al., 2021, Zhao et al., 2021).

## 6 Future perspectives

Despite remarkable advancements in the engineering of lignocellulolytic enzymes and the microorganisms that produce them, there is still a huge demand for future work to investigate and realize large-scale lignocellulose degradation in industry. For most published data in scientific literature to date, the production levels of lignocellulolytic enzymes by fungi are lower than the requirements (∼0.4 g protein/L/h at low costs) (Ellilä et al., 2017, Novy et al., 2019). In addition, the catalytic efficiency and inhibitor resistance of lignocellulolytic enzymes need to be improved in the context of specific biomass pretreatment strategies. To achieve these goals, extensive mining of the natural enzyme diversity and deep engineering of key enzyme components will have to be conducted. Furthermore, current studies on lignocellulolytic enzymes are highly fragmented and need to be integrated. As an example, engineered enzymes have rarely been expressed in industrial hosts. With the aid of efficient genome engineering methods (Liu et al., 2017, Zou et al., 2020), systematic genetic engineering is expected to produce highly active, robust, and well-proportioned lignocellulolytic enzyme mixtures at high levels.

## Declaration of Competing Interest

Given his role as Managing Editor, Dr. Guodong Liu, had no involvement in the peer-review of this article and has no access to information regarding its peer-review. Full responsibility for the editorial process for this article was delegated to Dr. Yuezhong Li.
